# Liver cirrhosis complicated with pulmonary *Nocardia* infection: A case report and literature review

**DOI:** 10.1097/MD.0000000000040054

**Published:** 2024-12-06

**Authors:** Yan Xiao, Hongwei Wang, Tingting Tian, Juanjuan Zheng, Mengyang Liu, Qing Wang, Jing Li

**Affiliations:** a Department of Clinical Laboratory, The Affiliated Hospital of Qingdao University, Qingdao, China; b Blood Transfusion Department, The Qilu Hospital of Shandong University Dezhou Hospital, Dezhou Shandong Province, China.

**Keywords:** case report, human infection, liver cirrhosis, *Nocardia*

## Abstract

**Rationale:**

*Nocardia* is a conditionally pathogenic bacterium found in the natural environment and is an aerobic bacterium, which causes severe pneumonia relatively rarely. The diagnosis of primary *Nocardia* infection is always made late due to the nonspecific clinical presentation of patients with *Nocardia* infection and the time required for *Nocardia* culture. Due to its rarity and nonspecific clinical presentation, patient survival is often compromised due to misdiagnosis.

**Patient concerns:**

A 53-year-old woman with autoimmune cirrhosis was treated with glucocorticoids. Three and a half months later the patient developed fever, nausea, cough and sputum, treated with empirical antibiotics, and the patient occurred type I respiratory failure.

**Diagnoses:**

*Nocardia* was identified by sputum culture micromorphology and Meriere matrix-assisted laser desorption ionization time-of-flight mass spectrometry (MALDI-TOF).

**Interventions:**

The patient was treated with trimethoprim-sulfamethoxazole (TMZ), and the patient’s oxygen saturation recovered significantly.

**Outcomes:**

The patient’s overall recovery was slow due to decreased albumin (ALB) and increased procalcitonin (PCT) caused by the patient’s cirrhosis. Due to economic reasons, the family gave up, asked to be discharged.

**Lessons:**

In our report, patients with underlying disease are immunocompromised and at increased risk of infection with conditionally pathogenic bacteria. *Nocardia* has no specific presentation and is often overlooked clinically. Treatment of these patients should not be limited to common bacteria or viruses, but should consider rare opportunistic pathogens, and we need to be vigilant for *Nocardia* infections and timely use of sulfonamide antibiotics to reduce mortality.

## 1. Introduction

*Nocardia* is a filamentous, aerobic, acid-fast gram-positive bacilli belonging to the order of actinomycetes. The diameter of *Nocardia* was 0.5 to 1.2 μm, and it was multidirectional branching filamentous, without spores or flagella. Abundant mycelium could be observed in the early stage of laboratory cultivating, often with secondary branching, and the bacteria would cleave into spherical or rod-shaped at the later stage of cultivating. More than 70 Nocardia species have been identified till now, of which 33 are pathogenic to humans,^[1]^ and the main pathogens includes *Nabscessus*, *Nnova* complex, *N farcinica*, *Nbrevicatena/Npaucivorans*, *Ntransvalensis complex*, and *Ncyriacigeorgica*.^[2]^ Among the diseases caused by Nocardia species, infection caused by *N farcinica* accounts for 24.5%, with a higher transmission and mortality.^[3,4]^

*Nocardia* is a saprophytic opportunistic pathogen, which could be widely found in air, soil and water.^[5]^Under normal circumstances, *Nocardia* is not pathogenic to immunocompetent individuals. Patients infected with *Nocardia* often have underlying diseases such as HIV infection, hematological malignancies, diabetes, liver cirrhosis, etc.^[6–8]^ Advanced age, use of hormone or immunosuppressive agents are also risk factors for *Nocardial* infection. *Nocardia* can cause infection in various parts, mainly in the lung, skin, central nervous system, cornea, spine and surgical incision.^[9]^ As the clinical symptoms of *Nocardia* infected patients are not specific, and the culture of *Nocardia* need a longer time, the primary *Nocardial* infection is often ignored or delayed, with the average time of 3 to 30 days to confirm the infection, which would lead to a disseminated infection finally.^[10]^ Here, based on clinical and laboratory work, we report a case of *Nocardia* infection in the lung, together with literature review, to provide new and early ideas for diagnosis and interventions of *Nocardia* infection.

## 2. Case report

**Figure 1.**
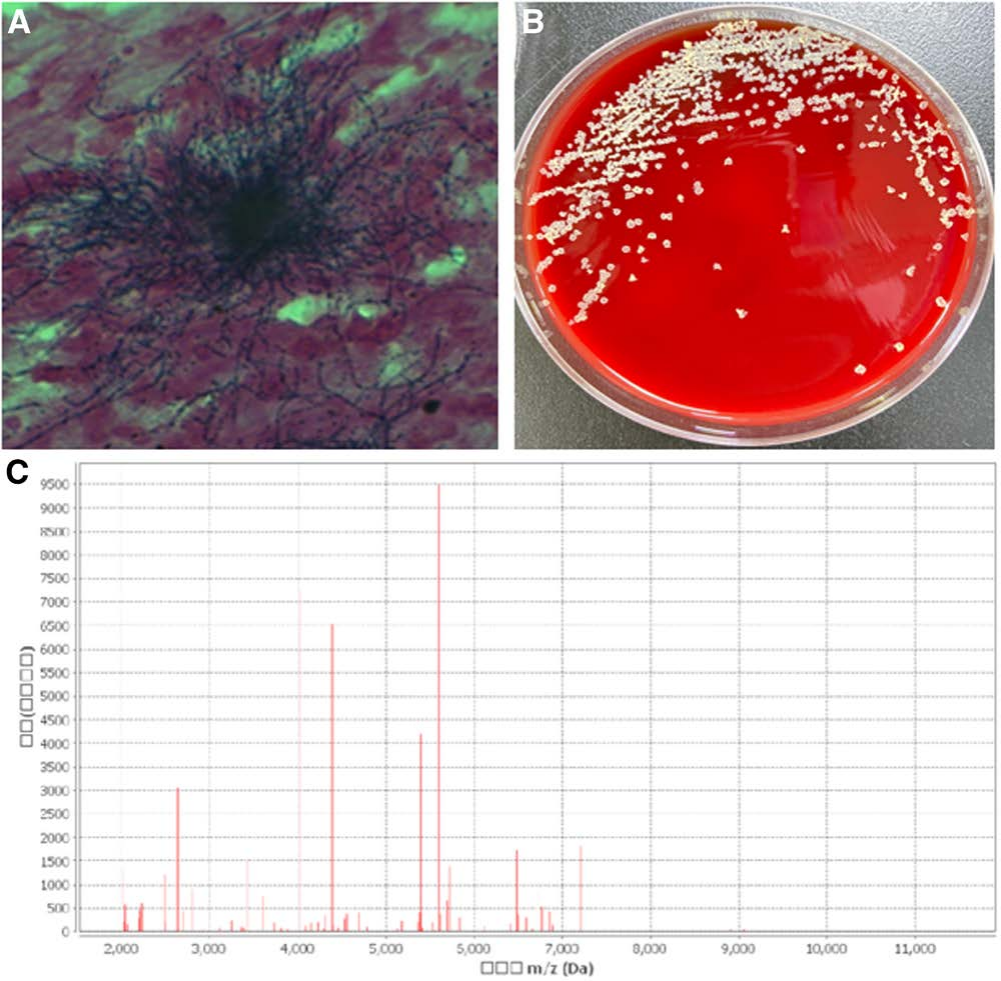
Sputum laboratory examination of this patient. (A) Gram staining of the sputum smear shows typical branched hyphae with a 90° branching angle. (B) After 7-day culture, Irregular colonies on Columbia blood agar plate appeared, taking on a powdery aerial mycelium body on the colony surface, which had an earthy smell. (C) Identification of mass spectrometry.

**Figure 2.**
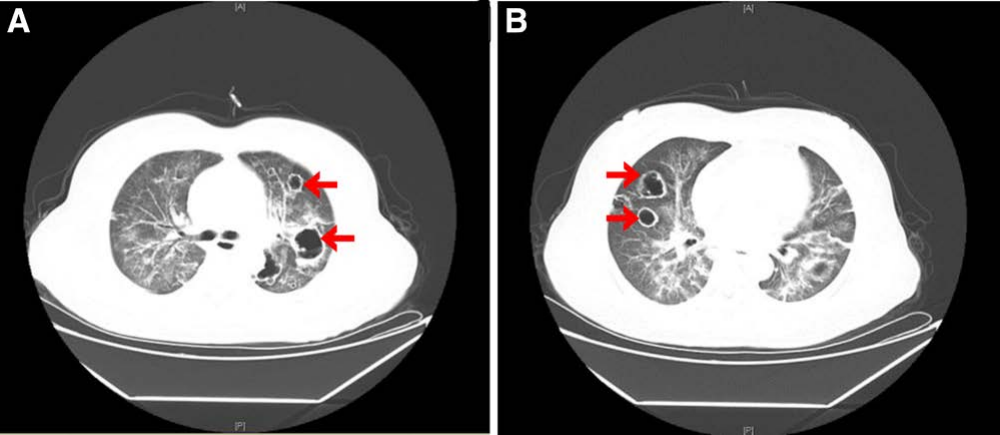
Computed tomography (CT) results of this patient. Chest CT of this patient showed multiple thick-walled cavities in both lungs (at the arrow mark), small amount of pericardial effusion; some mediastinal lymph nodes slightly enlarged, indicating a probability of inflammation of both lungs, not excluding mycobacterial infection.

**Figure 3.**
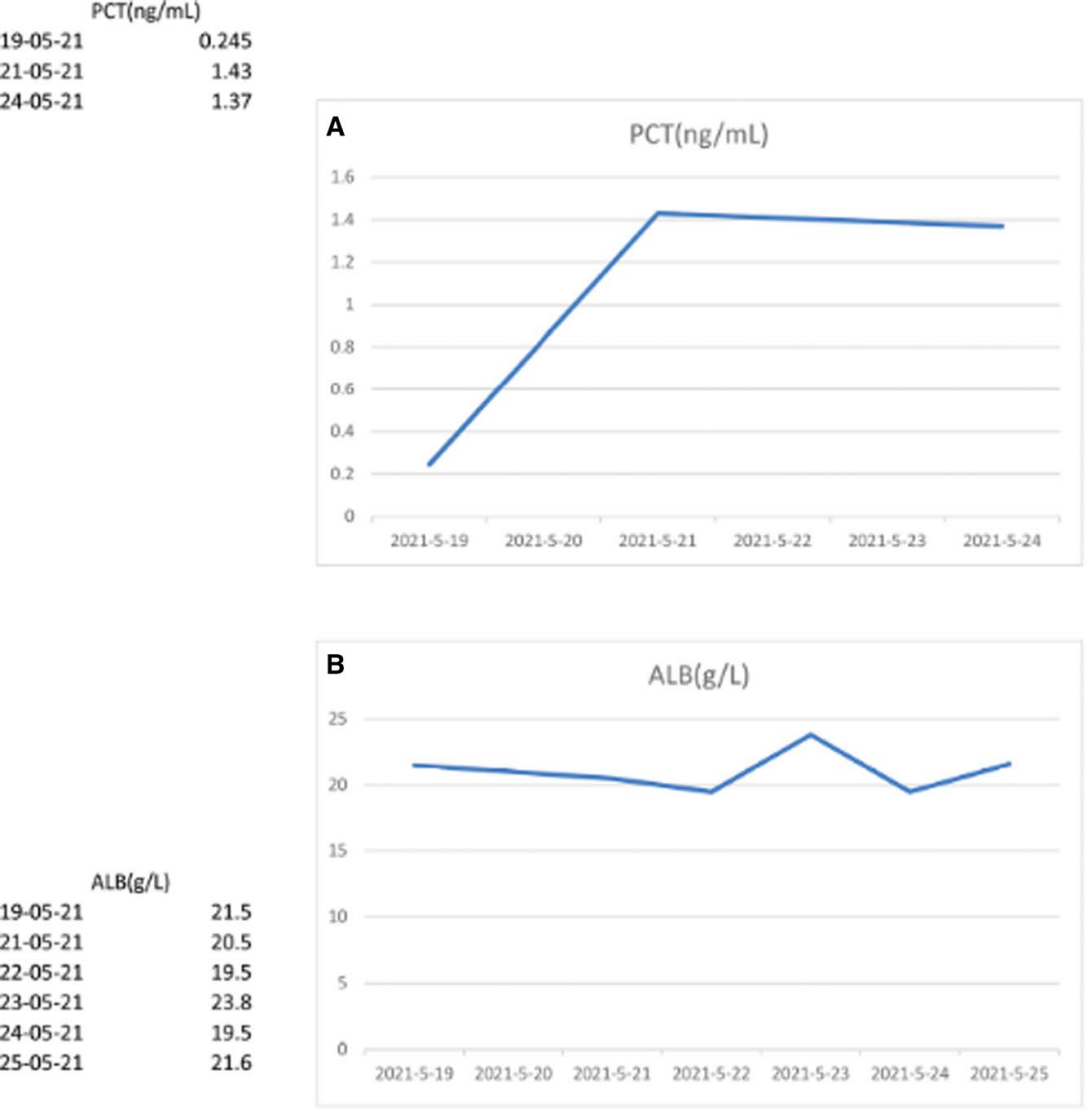
Blood laboratory examination of this patient. (A) Trends in patient PCT, always < 0.05 ng/mL after this hospitalization. (B) ALB much less than the normal number of 40 g/L.

A 53-year-old woman with an autoimmune cirrhosis was treated with glucocorticoids. After 2 and a half months of treatment, the patient’s skin and scleral jaundice were relieved, albumin levels increased, and aspartate aminotransferase (AST) and alanine aminotransferase (ALT) levels decreased. One month later, the patient developed fever and nausea, accompanied by symptoms of lung infection such as cough and sputum, and came to the hospital on 2021-05-18. After admission, blood routine test showed white blood cells 10.96 × 10^9^/L, neutrophil count 9.10 × 10^9^/L, lymphocyte count 1.03 × 10^9^/L, whole blood C-reactive protein (CRP) 28.66 mg/L, and procalcitonin (PCT) 0.245 ng/mL, and moxifloxacin was used for empiric anti-infection therapy. On the second day, laboratory examination showed that the patient’s fungal G (1, 3 β-D- glucan) test: 664.80 pg/mL, fungal GM (Galactomannan) test 0.67S/CO, gram stain of sputum smear showed a gram-positive bacilli with 90° branching angle hyphaes (Fig. 1A), and chest CT showed multiple thick-walled cavities in both lungs, not exclude mold infection (Fig. 2). Considering this, voriconazole was added to the therapy list. Two days later, on the 4th day, the patient’s fever and nausea were not significantly alleviated, indicating the treatment would not work well. The patient’s electrocardiogram monitoring showed that the pulse oxygen saturation was reduced to 60% to 70%, and the blood gas analysis (ABG) showed: PH value 7.48, lactic acid 3.10 mmol/L, carbon dioxide partial pressure 32.60 mmHg, oxygen partial pressure 53.00 mmHg, oxygen saturation 84.70%, oxygenated hemoglobin 82.90%, indicating a type I respiratory failure. Then the patient was transferred to the Intensive Care Unit (ICU), the antibiotic regimen was adjusted to voriconazole, imipenem combined with vancomycin, together with other supporting treatments such as liver protection, albumin supplementation, parenteral nutrition, and intestinal flora supplementation. On the 7th day after admission, sputum culture produced Irregular colonies, powdered aerial mycelium appearing on the surface of the colonies, colonies with earthy odor and producing yellow pigments. The gram-positive bacilli from the patients’ sputum culture (Fig. 1B) were identified as *Ncyriacigeorgica* by Meriere matrix-assisted laser desorption ionization time-of-flight mass spectrometry (MALDI-TOF), with the 99.99% identification percentage (Fig. 1C). The antibiotics were adjusted to voriconazole, imipenem combined with trimethoprim- sulfamethoxazole (TMZ) and caspofunging acetate (Table 1). Coordinate with other supported treatment such as mechanical ventilation, appropriate sedation and analgesia, the patients’ oxygen saturation rose to 90%. However,the patient’s albumin (ALB) had been always much lower than the normal range, and PCT did not decrease significantly (Fig. 3). Several weeks later, the family couldn't bear the cost and gave up, asked to be discharged.

**Table 1:** Use of anti-infective drugs during the patient’s hospitalization.

	5.18	5.19	5.20	5.21	5.22	5.23	5.24	5.25
MON	√	√	√	√				
IPM				√	√	√	√	√
VAN				√	√	√	√	
Fluconazole			√	√	√	√	√	√
SMX							√	√
CAS								√

Abbreviations: CAS = caspofungin, IPM = imipenem, MON = monensin sodium, SMX = sulfamethoxazole, VAN = vancomycin.

## 3. Review of literature

During the diagnosis and treatment of this patient, we investigated cases of *Nocardia* infection in our hospital retrospectively. The definite diagnosis for *Nocardia* infection is positive of *Nocardia* culture. From April 2013 to date, there have been 50 cases of *Nocardia* infection (Table 2). Among these patients, 43 had underlying diseases, which includes 19 with pulmonary diseases (5 cases of bronchiectasis, 4 cases of chronic obstructive pulmonary disease, 4 cases of tuberculosis, 2 cases of chronic bronchitis, 2 cases of asthma, 1 case of emphysema, and 1 case of lung cancer), 7 renal diseases (4 cases of nephrotic syndrome, 2 cases of kidney transplantation, and 1 case of chronic renal insufficiency), 7 systemic lupus erythematosus, 3 liver diseases (2 cases of chronic hepatitis B and 1 case of hepatic insufficiency). Besides, there were 21 patients who had previously used glucocorticoids or immunosuppressants. Most patients (37/50) had a good prognosis after treatment, with a total of 8 deaths and 5 discharged proactively.

**Table 2:** Clinical features of 50 patients with *Nocardia* infection in our hospital during 10 years.

Case	Sex	Underlying diseases	Special medications used	Specimen	Infectious bacteria	Symptoms	Imaging manifestations	Treatment	Prognosis
1	F	Systemic lupus erythematosus and Herpes zoster	Yes	Pleural fluid	*N farcinica*	Chest pain, fever, cough, sputum	Pleurisy with wrapped effusion, pneumonia, pleural thickening	SXT + MXF + CPZ	Survival
2	M	Herpes zoster, associated small vessel vasculitis	Yes	Sputum	Nocardia species, Cryptococcus	Body aches, weakness, recurrent fever	Pneumonia, multiple lymphadenopathy, pleural thickening	SXT + PIS + VOR+ Oseltamivir	Automatic hospital discharges
3	M	COPD	No	Sputum	Nocardia species, Pseudomonas albicans, Aspergillus genus	Intermittent feeling of suffocation	Multiple lesions in both lungs	Fluconazole	Survival
4	M	Atrial fibrillation	No	Sputum	*N farcinica*	Fever, chest tightness, breathlessness	Emphysema, double pneumonia	MER + PIS + LZD + SXT + Fluconazole	Death
5	M	COPD	No	Sputum	Pseudomonas albicans, Nocardia species	Cough, sputum, breathlessness	Bilateral branch enlargement with infection and lymphadenopathy	IMP + MXF + SXT + Fluconazole	Survival
6	M	Systemic lupus erythematosus	Yes	Sputum	*N farcinica*	Asymptomatic, for shock treatment	Multiple nodules and patchy shadows	CSL + Fluconazole + SXT	Survival
7	M	Chronic bronchitis	No	Sputum	Pseudomonas maltophilia, Nocardia species	Fever	Branch expansion and infection	Fluconazole + MX F + MNO	Survival
8	F	Hepatitis B	Yes	Sputum	Nocardia species	Cough and sputum, chest tightness, chest pain	Cavitated, patchy shadows	SXT + PIS + LVX	Survival
9	M	Hypertension, Nephrotic syndrome	No	Sputum	Pseudomonas aeruginosa, Nocardia species	Bilateral lower limb edema, fever	Fungal infection is likely	PIS + MNO	Survival
10	M	Tuberculosis	No	Sputum	Nocardia species	Fever, cough, sputum	Chronic bronchitis in both lungs with infection	MXF + SMX + AM K	Death
11	M	Bronchiectasis	No	Sputum	Nocardia species	Fever	Multiple infections, nodules	SXT + IPE + LZD	Survival
12	M	\	No	Sputum	Nocardia species, *Candida albicans*, Pseudomonas maltophilia	Cough and sputum, choking, fever	Branch lung enlargement and infection, multiple plaques, pleural thickening	SMZ + LVX	Survival
13	M	Asthma, Nephrotic syndrome, Hypertension	Yes	Blood	*N farcinica*	Fever, cough	Infection	CPZ + IMP + MER + SXT	Survival
14	M	Lymphoma	Yes	Pus in the brain	Nocardia species	Headache, low-grade fever	Brain abscess	AMK + IMP + CRO + SMX + LZD	Survival
15	F	Systemic lupus erythematosus	Yes	Sputum	Nocardia species	Fever, breathlessness	Infections	SXT + IMP	Survival
16	F	Tuberculosis, Coronary heart disease	No	Sputum	Nocardia species	Cough, coughing and sputum	Infection, tuberculosis and bacteria are more likely	CPZ + Oseltamivir + SXT	Survival
17	M	Cerebral thrombosis, diabetes mellitus, Emphysema	Yes	Sputum	Nocardia species	Weakness of the limbs	Emphysema, scattered in inflammatory nodules	SMX + VAN + CPZ + Fluconazole	Automatic hospital discharges
18	M	Chronic bronchitis, Chronic heart disease of pulmonary origin	No	Sputum	*Aspergillus fumigatus*, Nocardia species	Coughing, coughing up sputum	Double pneumonia	LZD + Fluconazole + AZM + PIS	Death
19	F	Gastritis, Bronchiectasis, Asthma	No	Sputum	*Streptococcus gramineus*, *Streptococcus desiccans*, Nocardia species	Cough, sputum, feeling of suffocation	Branches enlarged and infected	CPZ	Survival
20	F	\	No	Ear pus	Nocardia species	Pus in the right ear	\	Fluconazole + CAZ	Survival
21	M	Hypertension, Kidney transplant	Yes	Foot pus	Nocardia species	Infection of the left foot	\	PIS + CPZ + LZD	Survival
22	F	Systemic lupus erythematosus	Yes	Cerebrospinal fluid	Nocardia species	Fever	Double pneumonia	SMX + LZD + AM K	Death
23	M	Kidney transplant	Yes	Pleural fluid	Nocardia species	Chest pain	Multiple patchy opacities and nodular opacities	SMZ + TMP	Death
24	M	Bronchiectasis, Lung tuberculosis	No	Sputum	Nocardia species	Coughing and coughing up sputum and blood	Dilated and infected, chronically inflamed with right lung	MSU	Survival
25	F	Hypertension, Rheumatoid arthritis	Yes	Exudate from the sinus tract of the hip joint	Nocardia species	Pain in the left hip area due to a fall	\	CLI + IMP + AMK	Survival
26	M	Tuberculosis, Uroepithelial carcinoma	Yes	Alveolar lavage fluid	Nocardia species	Fever	Cancer of the right lung, double emphysema	LVX + CDR	automatic hospital discharges
27	F	Anca-associated vasculitis, Bronchiectasis	Yes	Sputum	*N farcinica*, Pseudomonas albicans	\	Branches enlarged and infected	BIA	Survival
28	F	Hepatitis B	No	Blood	Nocardia species	Loss of appetite, weakness and dizziness	Chronic inflammation	Unknown	Survival
29	M	\	No	Sputum	Nocardia species	Fever, cough, breathlessness	\	PIS + MXF + LZD	Death
30	M	Hypertension, Nephrotic syndrome	Yes	Skin in Pus	*N farcinica*	Cough, coughing and sputum	Multiple clumps in both lungs, nodular shadows with cavities	PIS + MNO + SMX + IMP + MER	Survival
31	F	Systemic lupus Erythematosus, Tuberculosis	Yes	Blood	*N farcinica*	Fever	Chronic inflammation of both lungs	SXT	Death
32	M	Hypertension, Systemic lupus erythematosus	Yes	Feces	*N farcinica*	Pain in left shoulder with sore throat	\	Fluconazole + FEP + MNO	Survival
33	F	Rheumatoid arthritis	Yes	Sputum	*N abscessus*	Fever	Chronic inflammation	\	Survival
34	M	Aplastic anemia	Yes	Cerebrospinal fluid	Nocardia species	Headache	\	LZD + MER	Survival
35	M	Diabetes	No	Sputum	*N farcinica*	Cough, chest tightness, loss of appetite	Infection, pleural thickening, lymphoma	Fluconazole	Survival
36	F	Gastritis	No	Sputum	*N farcinica*	Hemoptysis	Decreased right lung, slightly narrowed bronchi, inflammatory changes?	PIS	Death
37	F	Lung Cancer	No	Alveolar lavage fluid	*N farcinica*	Cough	Postoperative changes in the upper lobe of the left lung	LVX	Survival
38	F	Pemphigus, Hypertension	No	Pus in the brain	*N farcinica*	Headache	\	MOX + VAN + ME R + SMX	Automatic hospital discharges
39	M	Hypertension	No	Pus in the brain	*N farcinica*	Headache, fever	Right temporal lobe brain abscess	CRO + VAN + MER + SM X	Automatic hospital discharges
40	F	\	No	Sputum	*N farcinica*	Fever	Branches enlarged and infected	CDR	Survival
41	M	Dilated cardiomyopathy, Hypertension, Gout	No	Sputum	Nocardia species	Feeling of suffocation	Double pneumonia, emphysema	IMP + ITRAC	Survival
42	F	\	No	Sputum	Nocardia species	Haemoptysis	Branches enlarged and infected	CAZ	Survival
43	M	COPD, Nephrotic syndrome	Yes	Sputum	*N cyriacigeorgica*	Cough and sputum, fever	Double pneumonia	BIA + SXT + ITRA C	Survival
44	F	\	No	Sputum	Nocardia species	Cough	\	SMX	Survival
45	F	After mastitis surgery	No	Pus	Nocardia species	Fever	\	Cephalosporin + O RN	Survival
46	M	\	No	Pus	Nocardia species	Swelling in the limbs	\	MXF	Survival
47	M	Diabetes	No	Sputum	*N brasiliensis*	Cough with sputum and fever	Multiple nodules in both lungs, lumpy shadows, mediastinal lymphadenopathy	BIA + SXT	Survival
48	M	Chronic renal Failure, Systemic lupus erythematosus, Hypertension, Heart Failure	Yes	Sputum	*N asteroides, Pseudomonas tropicalis*	Cough with sputum and fever	Multiple nodular plaques thickened on the right pleura	SXT	Survival
49	M	Hepatic insufficiency	No	Alveolar lavage fluid	*N otitidiscaviarum*	Coma	Double pneumonia, pleural effusion	PIS + SMX + Fluconazole	Survival
50	M	Bronchiectasis, COPD	No	Alveolar lavage fluid	Nocardia species	Coughing, feeling suffocated	Infection, pleural thickening	SMX	Survival

Abbreviations: AMK = bumikana; AZM = azithromycin; BIA = biapenem; Special medications used = glucocorticoids or immunosuppressive drugs; CAZ = ceftalidime; CDR = cefdinir; CLI = clindamycin; CPZ = cefoperazone sodium-sulbacta sodium; CRO = ceftriaxone; CSL = cefoperazone-sulbactam; F = female; FEP = cefepime; IMP = imipenem–cilastatin sodium; IPE = imipenem/EDTA; ITRAC = itraconazole; LLVX = levofloxacin; LZD = linezolid; LZD = linezolid; M = man; MER = meropenem; MNO = minocycline; MOX = moxalactam; MSU = mezlocillin-sulbactam; MXF = moxifloxacin; MXF = moxifloxacin; ORN = ornidazole; PIS = piperacillin-sulbactam; SMX = sulfamethoxazole; SXT = cotrimoxazole; TMP = trimethoprim; VAN = vancomycin; VOR = voriconazole; \ = unknown’s = chronic obstructive pulmonary disease.

In addition, we conducted a literature review of nocardiosis cases in patients with cirrhosis. In December 2022, 81 relevant articles in all fields of PubMed were retrieved using “*Nocardia* and cirrhosis” as search terms. We excluded cases of *Nocardia* infection in patients with cystic fibrosis,^[11]^ cirrhosis after liver transplantation and 1 suspicious case of an unspecified pneumoniae, and there were 10 papers left related to this topic^[12–21]^ (Table 3). Middle-aged or elderly women are the main group affected, which may be due to the lower immunity of them. The prognosis of nocardiosis after liver cirrhosis is poor and the mortality rate is high. Main pathogenic bacteria strains for *N farcinica*, reported cases of the most populous country is Japan. Some cirrhosis has a relatively clear cause, such as alcoholic cirrhosis or Viral hepatitis. The main site of *Nocardia* infection after cirrhosis is the lungs, which is consistent with the common site of *Nocardia* infection without underlying disease, and the 2nd easily infected organ is the brain. Most patients were treated with antibiotics, and sulfamethoxazole was usually the first choice. However, for patients with normal immune function, the treatment effect of sulfamethoxazole is good, while for patients with liver cirrhosis, lower immunity often accompanied, the effect would not be so good, and the poor outcome, drug resistance and toxicity would appear. A 79-year-old patient with cirrhosis and sarcoidosis was admitted to hospital and presented with respiratory distress and confusion, which was poorly treated with antibiotics and home oxygen therapy. The patient’s autopsy report indicated that *Nocardia* was cultured in the brain abscess. The cause of dyspnea is hepatopulmonary syndrome (HPS), that is, abnormal dilation of blood vessels in the lungs, gas exchange disorders, arterial oximetry abnormalities, and a series of pathophysiological changes and clinical manifestations on the basis of chronic liver disease or portal hypertension. In patients with sarcoidosis with liver involvement, abnormal gas exchange is disproportionate to the extent of lung involvement, and the possibility of HPS should be considered. The patient’s confusion may be due to a brain abscess from paradoxical embolism, and his cirrhosis and sarcoidosis decreased his immunity, which also increased the possibility of opportunistic infections. Autopsy brain slices showed focal cerebral hemorrhage, local cerebral vasculitis, and bacterial meningitis, and the characteristics of bacterial microbial colonies were observed, which were more consistent with *Nocardia*.^[16]^ A retrospective investigation of culture-confirmed *Nocardial* infection in patients with invasive nocardiosis in the National Taiwan University Hospital during the past 18 years was conducted. Occurrence of spontaneous pericarditis with *Nocardial* peritonitis infection in a patient with liver cirrhosis. Results showed that the immune function of patients with liver cirrhosis was low, which increased the possibility of spontaneous inflammation and caused *Nocardial* opportunistic infection.^[14]^

**Table 3:** PubMed articles related to this topic.

Case	Sex	Age	Reporting countries	Underlying diseases	Special medications used	Causes of cirrhosis	Location of the infection	Specimen	*Nocardia*	Symptoms	Imaging manifestations	Treatment	Prognosis
1	F	67 y	United Kingdom	Diabetes mellitus, Liver cirrhosis	Corticosteroids	Unknown	Lung	Sputum	*Nocardia caviae*	Fever, cough, green sputum, malaise, weight loss	Patchy consolidation in lungs, relative sparing of the apices	Gentamicin 60 mg 8 hourly, sulphadimidine 500 mg 6 hourly for 6 mo, sulphadimidine 2 g/d for a further 3 mo	Improve
2	F	84 y	Japan	Liver cirrhosis	Unknown	Hepatitis C	Chest wall	Pus	*N farcinica*	Progressive bulging of the anterior chest wall for 2 weeks	7 cm × 7 cm × 5 cm loculated empyema, rib destruction	Trimethoprim/sulfamethoxa zole 5 mg per kg per dose 3 times a day and subcutaneous debridement	Improve
3	Unknown	Unknown	Taiwan, China	Liver cirrhosis	Unknown	Unknown	Spontaneous nocardial peritonitis	Unknown	Nocardia species	Unknown	Unknown	Unknown	Unknown
4	F	79 y	Canada	Sarcoidosis, Cirrhosis with hepatopulmonary syndrome (HPS)	Corticosteroids	Typical sarcoid - type granulomatous hepatitis	Brain	Brain sections	Nocardia species	worsening dyspnea 3 days, weakness confusion	chest x-ray was unremarkable	Unknown (treated for a non-ST elevation myocardial infarction)	Death
5	M	59 y	Australia	Cirrhosis of the liver, chronic obstructive pulmonary disease	Unknown	Hepatitis C-related liver cirrhosis	Abdomen	Ascites	Nocardia species	Non-colicky abdominal pain 2 weeks	Oedematous liver, ascites, port o-systemic varices	Amikacin 1500 mg daily and meropenem 2 g twice daily	Death
6	F	78 y	Japan	Cirrhosis of the liver, Esophageal varices, Hypertension, Gallstones	Unknown	Unknown	Brain	Pus	*N farcinica*	Fever, incomplete hemiparesis and mild dysarthria	Lesion in the right frontal lobe. Infiltrative shadow in lung.	Perforated drainage, sulfamethoxazole/trimethoprim × 1 2 g/d, pazufloxacin (PZFX) minocycline × 1 g/d, meropenem × 1 g/d, linezolid (LZD) × 1200 mg/d, amikacin × 400 mg/d	Death
7	M	27 y	Spain	Cirrhosis and Human Immunodeficiency Virus Infection, Tricuspid endocarditis, Acute hepatitis	Unknown	Alcoholic cirrhosis of the liver	Abdomen	Ascitic fluid	*Nocardia asteroides*	Reaccumulation of ascites	Unknown	Cefotaxime and cotrimoxazole × 12 d, sulfadiazine (6 g/d)	Death
8	M	56 y	Japan	Diabetes, Cirrhosis of the liver	Unknown	Alcoholic cirrhosis of the liver	Brain	Pus	*N farcinica*	Headache, general weakness, dysarthria	Rosaceous low signal area in the cerebellar hemisphere, brain cadre edema	Sulfamethoxazole/trimethoprim, Craniotomy for abscesses	Improve
9	F	49 y	United States	Liver cirrhosis	Unknown	Alcoholic liver cirrhosis	Pleura	Pleural effusion	*N farcinica*	Fever, dyspnea and right pleuritic chest pain of 1-wk duration	Ground – glass opacification, pleural effusion and ascites	Cefotaxime, two thoracenteses of 600 mL each; Cefotaxime was replaced by linezolid 600 mg po q12h 1 mo, Ciprofloxacin (500 mg po q12 h) 3 mo	Survived nocardiosis (succum bed to other complications)
10	F	71 y	\	Cirrhosis, Hypoalbuminemia and ascites	Unknown	Alcoholic liver cirrhosis	lung	Lung tissue	*Nocardia*	Lower lobe pneumonia and pleural effusions	Unknown	Trimethoprim and sulfamethoxazole	Lost to follow-up

## 4. Discussion

In this study, we reported a case of *Nocardia* infection in cirrhosis after autoimmune hepatitis, who take glucocorticoids and immunosuppressants to improve her jaundice symptoms and liver function. However, the autoimmune hepatitis and the glucocorticoidse use weakened her immunity and increased the possibility of opportunistic infections. The patient developed *Nocardia* infection in her lung. The gram stain and culture of sputum were performed in our laboratory, and the gram-positive bacilli with branched bodies in the sputum was detected on the second day, which was identified as *Nocardia* later on 7 days. Upon this case, we collected all *Nocardia* cases of our hospital and found that 86.0% of patients had underlying diseases, among which lung diseases accounted for 44.1%, renal diseases, systemic lupus erythematosus and liver diseases each for 16.2%, 16.2%, 7.0 % respectively. Treatment with sulphonamides was conducted in 52.0% of patients and 69.2% of them improved. 42.0% of patients had been on glucocorticoids or immunosuppressive drugs and16.0% of them died. Patients with no previous underlying disease had better outcome, with 85.7% of them improved. Of the deaths in our patients with *Nocardia* infection, 38% had pulmonary disease and 25% had systemic lupus erythematosus. Besides, 38% of the death had previously glucocorticoidse or immunosuppressive drugs use. The dose of glucocorticoids or immunosuppressive drugs significantly reduces the body’s immune capacity and increases the risk of *Nocardia* infection, which could easily lead to progression of the underlying disease in return, amplify the toxicity of drug and cause a high mortality. This suggests that drug uses need to be considered the benefits and risks associated. For patients with autoimmune hepatitis, the use of glucocorticoids and immunosuppressive drugs can inhibit damage to the liver and is a common clinical treatment option, but there is still a risk of poor treatment outcomes and progression to cirrhosis. Patients with cirrhosis have reduced levels of bile and pancreatic digestive enzymes due to impaired gallbladder and pancreas function, which can directly affect the digestion and absorption of fats and proteins. Patients also tend to have a poor appetite and poor nutrition, which can lead to low collective protein synthesis and poor immunity, increasing the risk of *Nocardia* infection. Patients have impaired liver function and a reduced ability to metabolize sulphonamides, exacerbating the toxicity of drug use and leading to a poor prognosis. This is more consistent with our review of the literature, which reports a poor prognosis for *Nocardia* infection in patients with cirrhosis, with a mortality rate of 40%. A patient with *Nocardia* infection survived treatment but had a significant adverse reaction to the antibiotics used during treatment and later died of other infectious diseases.

At present, sulfonamides are still the first choice for the treatment of nocardiosis, although in the study of Larruskain et al, the resistance rate to cotrimoxazole was 41.7%.^[22]^ Identification and culture of *Nocardia* are difficult. Molecular methods, especially gene sequencing, have become the most accurate and fast method to identify *Nocardia* at the species level and 16S rRNA, secA1, HSP65, gyrA, and rpoB were the most commonly used genes.^[23]^

*Nocardia* is gram-positive, aerobic, and a rare conditional pathogen. Due to the low rate of infection, it is easily missed clinically, and patients do not receive targeted treatment, thus the prognosis is usually poor. Timely administration of sulfonamides can reduce mortality in patients with nocardia. *Nocardia* has no specific presentation and is often overlooked clinically, leading to unclear diagnosis and delayed treatment, which places a significant prognostic and socioeconomic burden. Patients with Nocardia concentrate in the middle-aged and elderly population may be because the population often has underlying diseases or low immunity, while drugs used to treat underlying diseases, such as glucocorticoids or immunosuppressive drugs, cause liver function damage and increase the risk of opportunistic pathogenic bacteria. This suggests that when middle-aged or elderly patients present with fever, chest tightness and imaging reports suggesting no obvious specific changes such as inflammation, plaques or cavities in both lungs, they need to pursue the patient’s past medical history and medication use, and that treatment of infections in immunocompromised patients should not be limited to common bacteria or viruses, but should also consider rare opportunistic pathogens. For the treatment of immunocompromised patients, clinicians need to consider not only common bacterial or viral infections, but also rare opportunistic infections to maximize early diagnosis, targeted therapy, and cure rates.

## Author contributions

**Conceptualization:** Jing Li, Qing Wang.

**Data curation:** Tingting Tian, Juanjuan Zheng, Mengyang Liu.

**Writing – original draft:** Yan Xiao, Hongwei Wang.

**Writing – review & editing:** Jing Li.
